# Dermatophytosis beyond *Trichophyton indotineae*: genomic characterization of rare keratinophilic fungi *Myriodontium keratinophilum* and *Aphanoascus verrucosus* in North India

**DOI:** 10.1128/spectrum.01087-26

**Published:** 2026-09-25

**Authors:** Amtoj Kaur, Sandhyarani Mahanto, Gulnaz Bashir, Meenakshi Malik, Bashir Ahmad Fomda, Farah Sameem, Arijit Pal, Syed Khurshid Ahmad, Hardeep Kaur, Perumal Vivekanandan, Anuradha Chowdhary

**Affiliations:** 1 Medical Mycology Unit, Department of Microbiology, Vallabhbhai Patel Chest Institute, University of Delhi 72916 , Delhi, India; 2 Kusuma School of Biological Sciences, Indian Institute of Technology Delhi 28817, New Delhi, India; 3 Department of Microbiology, Sher-i-Kashmir Institute of Medical Sciences 29078, Soura, Jammu and Kashmir, India; 4 Department of Zoology, Ramjas College, University of Delhi 28742, Delhi, India; 5 Department of Dermatology, STD & Leprosy, Sher-i-Kashmir Institute of Medical Sciences - Medical College Hospital 29078, Bemina, Jammu and Kashmir, India; 6 Department of Microbiology, Sher-i-Kashmir Institute of Medical Sciences - Medical College 29078, Bemina, Jammu and Kashmir, India; 7 National Reference Laboratory for Antimicrobial Resistance in Fungal Pathogens, Vallabhbhai Patel Chest Institute 72916 , Delhi, India; Icahn School of Medicine at Mount Sinai, New York, New York, USA

**Keywords:** dermatophytosis, *Myriodontium keratinophilum*, antifungal susceptibility, genome, *ab initio*, itraconazole resistance, *Apinisia keratinophilum*

## Abstract

Fungal skin infections represent the fourth most prevalent disease globally, predominantly attributed to dermatophyte species such as *Trichophyton*. Nevertheless, sporadic instances of dermatophytosis may also arise from opportunistic, uncommon fungi. This study documents the association of keratinophilic fungi, *Myriodontium keratinophilum* and *Aphanoascus verrucosus*, with cases of dermatophytosis in Kashmir, North India. Internal transcribed spacer region sequencing of 64 isolates obtained from skin, hair, and nail samples of 62 patients identified 61% of isolates (*n* = 39) as *M. keratinophilum*, followed by *Trichophyton indotineae* and *A. verrucosus* in 20% (*n* = 13) and 19% (*n* = 12) cases, respectively. Furthermore, the first hybrid *de novo* genome assembly of *M. keratinophilum* is reported in the present study. Genomic analysis of both *M. keratinophilum* and *A. verrucosus* species revealed median intra-species single nucleotide polymorphism (SNP) differences of 100 and 27, respectively, suggesting highly related strains in the patient population. *De novo* functional annotation identified a higher number of coding regions in *M. keratinophilum* compared to *T. indotineae* and *A. verrucosus*, including carbohydrate-active enzymes (CAZymes) exhibiting unique substrate affinities and subtilisin 3 (*SUB3*) gene, previously implicated as a potential virulence gene in dermatophytes. Notably, in addition to all the multidrug transporters encoded by *Trichophyton* species, *M. keratinophilum* genome contains six putative ATP-binding cassette (ABC) transporters showing 40%–70% sequence similarity with the *CDR1* gene. *M. keratinophilum* exhibited reduced susceptibility to multiple antifungals, including high minimum inhibitory concentrations (MICs) (>16 mg/L) against griseofulvin and itraconazole. Importantly, itraconazole is a primary therapeutic agent for terbinafine-resistant *T. indotineae* infections, underscoring the necessity for early and accurate identification and intervention of dermatophytic infections.

**IMPORTANCE:**

The study reports cases of dermatophytosis associated with two rare keratinophilic fungi, i.e., *Myriodontium keratinophilum* and *Aphanoascus verrucosus*. The cases mainly presented as tinea corporis and cruris, commonly caused by *Trichophyton indotineae* in the Indian subcontinent, which may remain undiagnosed due to a lack of molecular identification. Furthermore, the first-ever genome report of *M. keratinophilum* and its *ab initio* functional annotation is useful for further studies and management of opportunistic pathogens that exhibit reduced susceptibility towards itraconazole, the first-line drug of treatment for terbinafine-resistant *T. indotineae* infections. An early correct molecular identification of the etiologic agents of skin infections is warranted to tackle the new agents of dermatophytosis at the earliest stage.

## INTRODUCTION

Dermatophyte fungal infections, commonly referred to as tinea, or ringworm, constitute the most prevalent dermatophyte skin infections. WHO estimates, based on the IHME’s GBD 2024 report, indicate approximately 650 million fungal skin infections worldwide, of which about half are dermatophytosis, with the incidence rate showing a steady upward trend [https://www.who.int/news-room/fact-sheets/detail/ringworm-(tinea), accessed on 1 May 2026]. Furthermore, economic costs associated with superficial fungal infections are high, as reported by the Centers for Disease Control and Prevention, U.S.A., the direct medical cost caused by these infections being approximately USD 845 million in 2019, with dermatophytosis accounting for over half of the outpatient visits (1). The worldwide increased incidence of superficial fungal infections, with a growing trend of resistance, has been a matter of global concern. These infections are primarily attributed to dermatophytes, a widespread and varied group of keratinophilic filamentous fungi that can invade human skin, hair, and nails. *Trichophyton* species represent the leading cause of dermatophytosis worldwide. Over the past decade, a novel dermatophyte species, *Trichophyton indotineae*, which exhibits resistance to first-line treatments, such as terbinafine, has been associated with an ongoing epidemic of challenging-to-treat disease in the Indian subcontinent (2, 3). *T. indotineae* is now a common infection worldwide that presents with widespread skin lesions often affecting several body sites. The highly inflammatory pruritic lesions are common on the groin, thighs, pubic/genital area, buttocks, and the face (4–6), usually manifesting as chronic, recurrent, or recalcitrant disease. Conventional diagnostic methods are inadequate for distinguishing *T. indotineae* from other closely related species, necessitating advanced molecular techniques, such as sequencing, for accurate differentiation (7). The emergence of this new species highlights the need for molecular identification and surveillance of dermatophytic infections to track the emergence of new infectious agents and their resistance profiles. Here, we report two rare keratinophilic fungi, *Myriodontium keratinophilum* and *Aphanoascus verrucosus,* associated with dermatophytic infections. Both species were increasingly isolated from skin, nail, and hair lesions of patients with dermatophytosis in the Kashmir region of North India. Furthermore, we report the isolation of other keratinophilic fungi, i.e., *Aphanoascus terreus*, from the environmental samples of public gardens, suggesting their close association with human surroundings. *M. keratinophilum* was first isolated and identified by Samson and Polonelli in 1978 from soil mycoflora in Italy (8). Since then, it has been reported from river sediments in Spain, soil from Maharashtra, India, and Korea, and from a chemoautotrophic cave in Romania (9–12). It has also been isolated from pigeon feathers in India, and Eurasian teal feathers in Tuscany (13, 14). Furthermore, isolation of *M. keratinophilum* from sea cucumber, mole cricket, and the textile remains of the funeral clothes of Cardinal Pázmány, an important historic figure who died in 1637 (Bratislava, Slovakia), suggests its association with diverse ecosystems (15–17). Recently, *M. keratinophilum* has been proven to be an entomopathogenic fungus infecting eight different pest species, including four agricultural and forest pests, such as oak lace bug and Colorado potato beetle, proving to be a potential insecticide with 30%–100% mortality among infected pests (18). The first human infection by *M. keratinophilum* was documented in 1985 in a 53-year-old Nigerian man with frontal sinusitis (19). Subsequently, it was also reported from the skin of a Polish resident (20) and from a case of keratomycosis (21). However, a definitive identification was lacking as no molecular characterization was done in any of these cases. Recently, Li et al. (22) isolated *M. keratinophilum* from mangrove sediments in China and renamed it as *Apinisia keratinophilum* (22). The isolate was identified based on the internal transcribed spacer (ITS) region sequencing, which aligned closely in the phylogenetic tree to *Apinisia pyriformis,* a species that was identified in the same study. However, the new nomenclature has not been updated in the NCBI database. Therefore, the old nomenclature of *M. keratinophilum* is retained in the present study.

The other rare agent of dermatophytosis, *A. verrucosus,* was first identified by Cano and Guarro in 1990 from a soil sample in Spain (23). *Chrysosporium tropicum* was the heterotypic synonym of *A. verrucosus*; however, in a recent update of the order Onygenales, Kandemir et al. merged the two species using multi-gene phylogeny and retained the name *A. verrucosus* (24). Furthermore, in a study from the Czech Republic, both the species *M. keratinophilum* and *C. tropicum* were isolated from nail infections (25). Previously, *A. verrucosus* (reported as *C. tropicum*) has been isolated from comb lesions of chickens in India and has been identified as a probable cause of mycosis in poultry (26). In sum, only a few sporadic case reports of *M. keratinophilum* and *A. verrucosus* in human infections have been documented. Here, in this study, we document a series of cases of dermatophytosis associated with *M. keratinophilum* (*n* = 39; 61%) and *A. verrucosus* (*n* = 12; 19%) among patients attending the outpatient department of dermatology in one of the largest medical institutes in Srinagar, Jammu and Kashmir, India. Interestingly, a few cases were linked to the classical agent of dermatophytosis, *T. indotineae* (*n* = 13; 20%). Importantly, most of the *M. keratinophilum* isolates exhibited reduced susceptibility to multiple antifungal agents. Furthermore, to understand the genomic insights of these keratinophilic fungi, we compared whole-genome assemblies of *M. keratinophilum* (*n* = 8), *A. verrucosus* (*n* = 7), and *T. indotineae* (*n* = 3) and further annotated the genomes of all three species.

## RESULTS

### Molecular identification of clinical isolates

**Fig 1:**
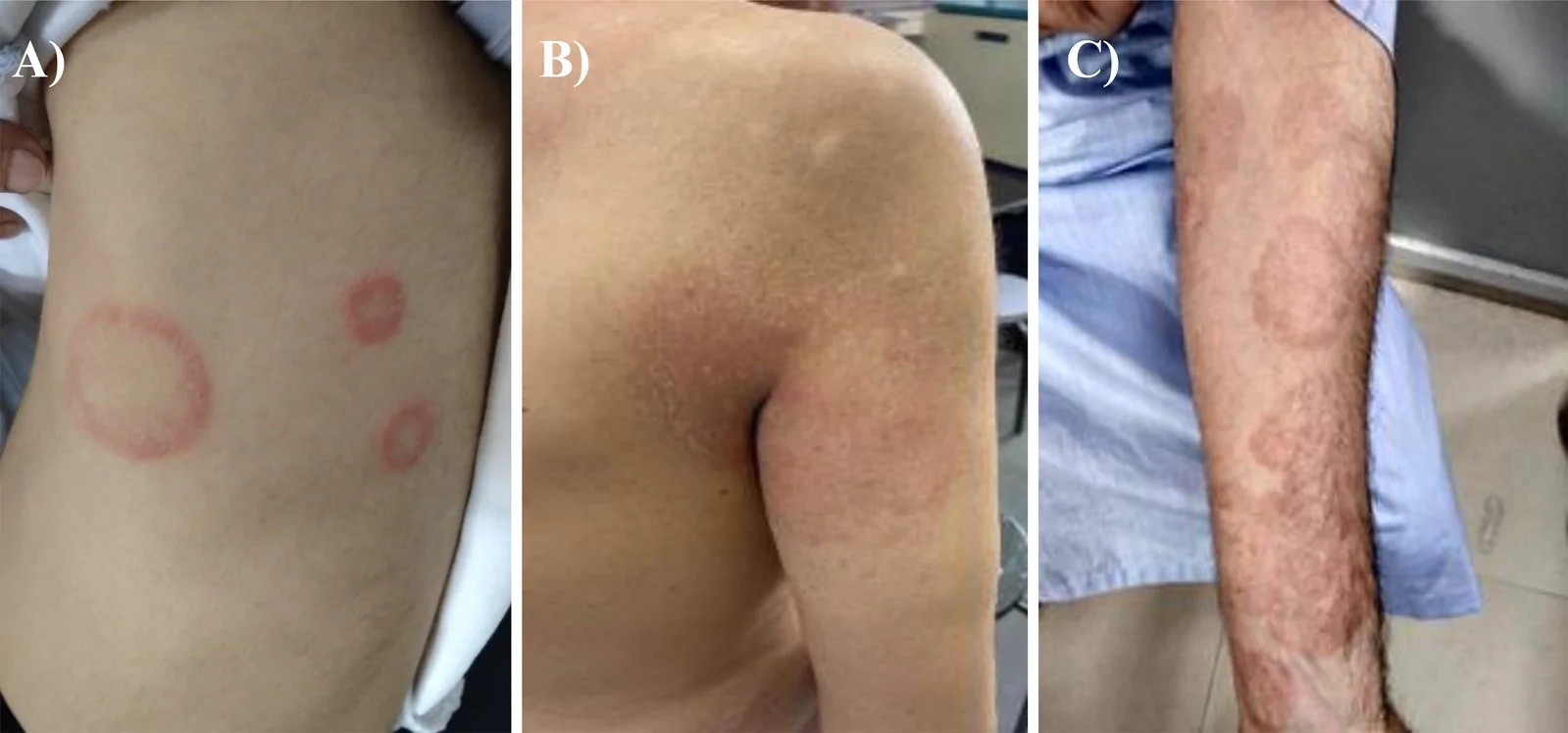
Tinea corporis lesions in patients positive for (**A**) *Myriodontium keratinophilum*, (**B**) *Trichophyton indotineae*, and (**C**) *Aphanoascus verrucosus*.

**Fig 2:**
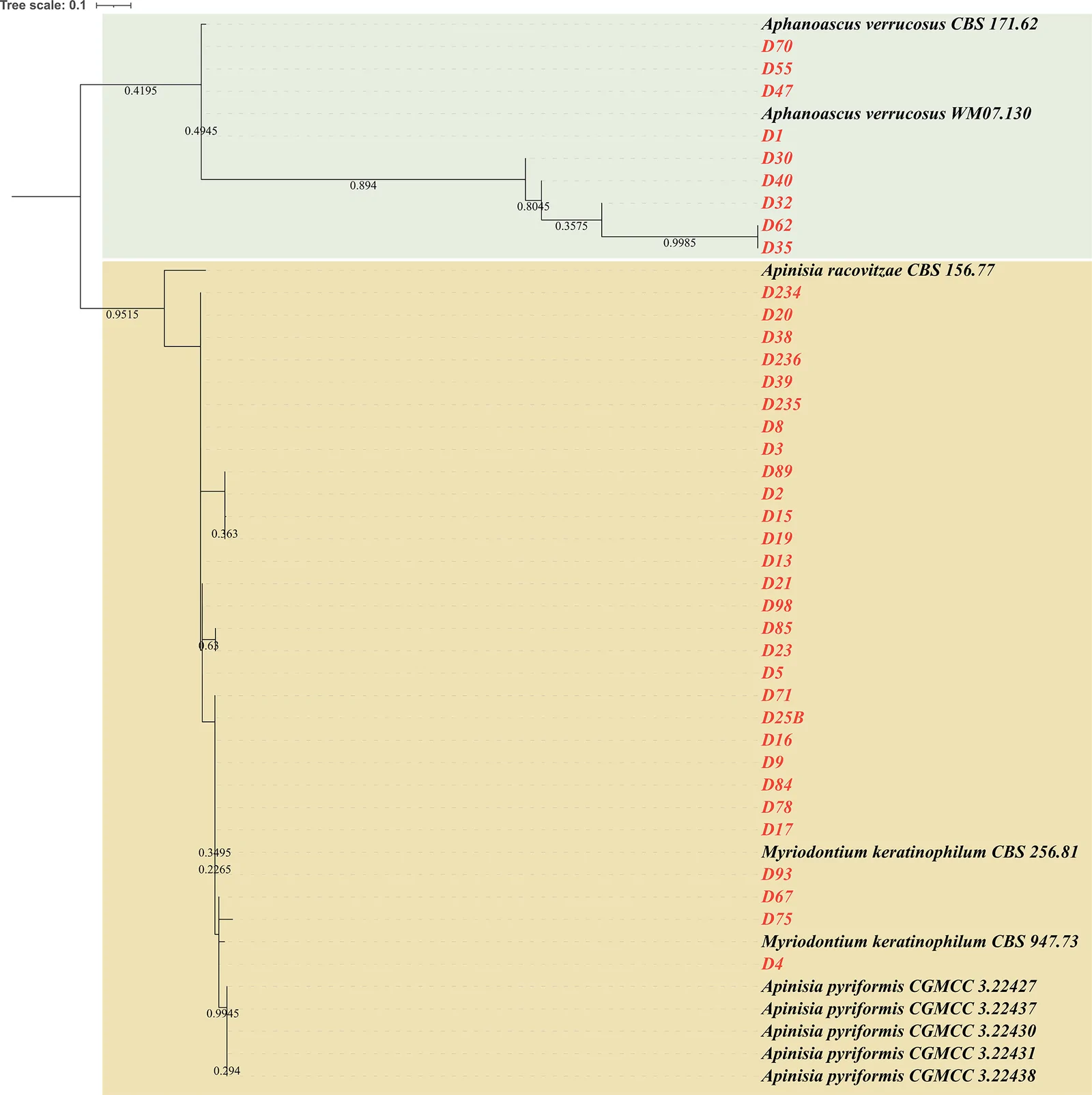
Phylogenetic tree based on the ITS region sequence: *M. keratinophilum* and *A. verrucosus* isolates sequenced in the present study are shown in red, and the reference strains (CBS, CGMCC, and WM07.130) are shown in black. Branch values (0.2–1) indicate the bootstrap (200–1,000) of the respective clade.

A total of 109 samples of skin scrapings, nails, and hair, collected from 107 patients with clinically suspected lesions of dermatophytosis attending the dermatology outpatient department during a period of 12 months (January to December 2021), were sent for processing in the mycology laboratory. Out of the 109 samples, 70 samples tested positive for fungal hyphae by direct microscopy of samples digested in 10% (40% for nail samples) potassium hydroxide (KOH) mount (Fig. S1) and grew in culture. Furthermore, eight samples were positive for fungal hyphae in KOH mount but yielded no growth in culture, whereas five samples yielded growth in culture but were negative in KOH mounts. In this study, we characterized all the fungal isolates (*n* = 70) obtained from patients with positive KOH mount. These isolates were identified by ITS region sequencing as *M. keratinophilum* (*n* = 39), *T. indotineae* (*n* = 13), *A. verrucosus* (*n* = 12), *Microsporum canis* (*n* = 3), *Trichophyton rubrum* (*n* = 2), and *Trichophyton violaceum* (*n* = 1). Only the former three species, including the two keratinophilic fungi and the well-established dermatophyte, *T. indotineae*, which together presented 91% (*n* = 64) of the total isolates, were included in the present study. The clinical distribution of these 62 patients was predominantly tinea corporis/cruris (Fig. 1; 50%, *n* = 32), followed by tinea capitis (17%, *n* = 11), and onychomycosis (16%, *n* = 10). Furthermore, seven patients presented with tinea facei (11%), and a single case each of tinea manuum and tinea incognito was recorded. Of these 64 isolates, 61% (*n* = 39) were identified as *M. keratinophilum*, followed by *T. indotineae* and *A. verrucosus* in 20% (*n* = 13) and 19% (*n* = 12) cases, respectively. Overall, 70% (*n* = 45) of the isolates were derived from skin samples, followed by nail (16%, *n* = 10), and hair (14%, *n* = 9). ITS-based phylogenetic analysis (Fig. 2) showed *M. keratinophilum* and *A. verrucosus* form separate clusters along with their respective reference strains, i.e., China General Microbiological Culture Collection Center (CGMCC) strains and/or Centraalbureau voor Schimmelcultures (CBS) strains, detailed in Table S1.

### Environmental sampling of dermatophytes in public parks

The environmental survey of soil samples and surface swabbing of benches and swings from public gardens yielded colonies of keratinophilic fungi *Aphanoascus terreus* from swing samples plated on dermatophyte test media (DTM). ITS region-based phylogenetic analysis of the environmental *A. terreus*, along with the clinical isolates of *A. verrucosus* from the present study, including the reference CBS strains and the type strains of other *Aphanoascus* spp., clustered all in the same clade (Fig. S2). Furthermore, *M. keratinophilum* isolates were placed separately in a different clade.

### Comparison of macroscopic and microscopic morphology of *M. keratinophilum, T. indotineae,* and *A. verrucosus*

**Fig 3:**
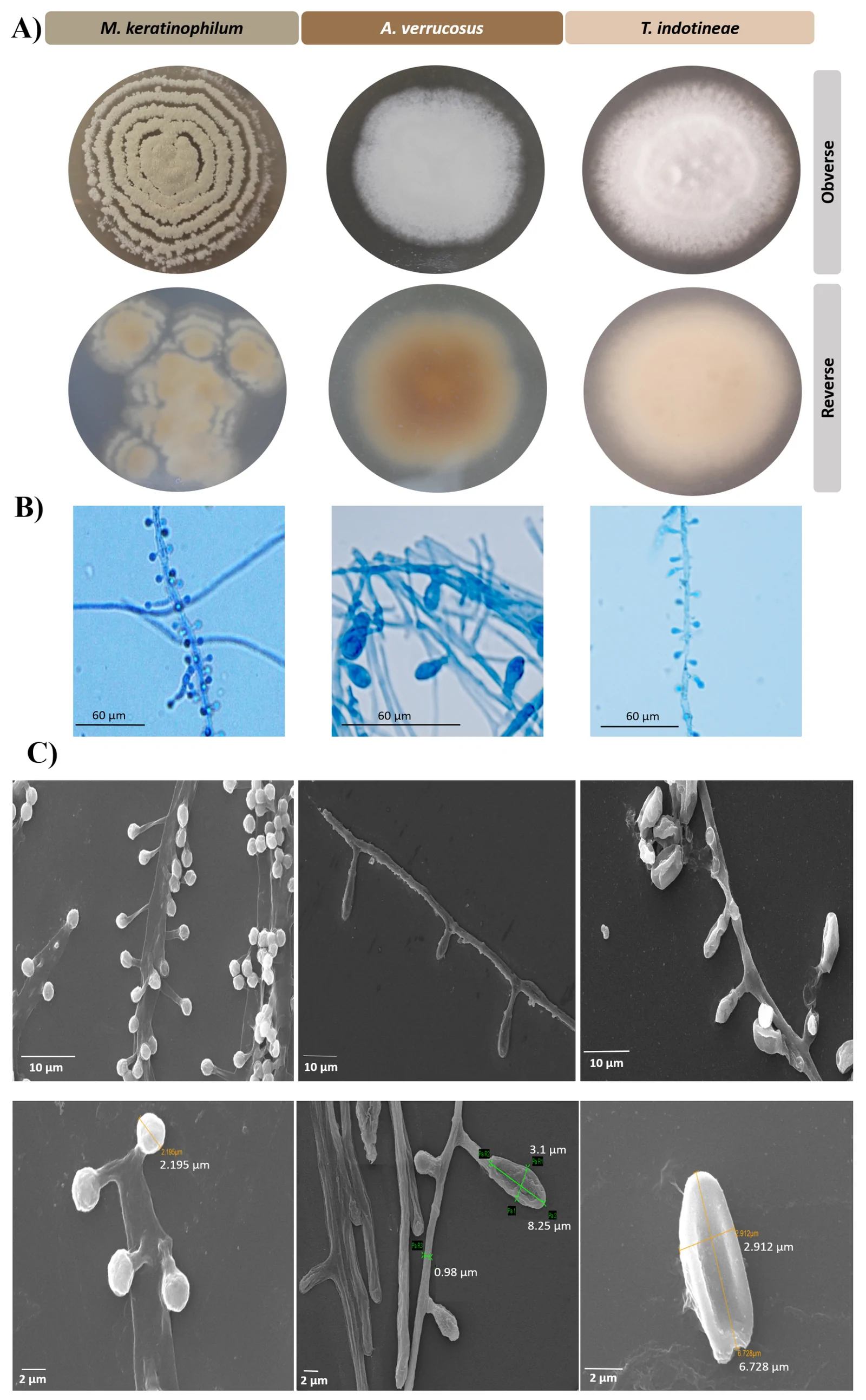
Morphological characteristics of *M. keratinophilum*, *A. verrucosus*, and *T. indotineae* on PDA after 7–10 days of growth. (**A**) Colony morphologies of the three species (obverse and reverse). (**B**) Lactophenol cotton blue staining of slide culture mounts (40×). (**C**) Microscopic morphologies captured using scanning electron microscopy.

The colony morphology of *M. keratinophilum* on potato dextrose agar (PDA) was powdery and buff with yellow coloration on the reverse after 7–10 days of incubation at 28°C. In contrast, colonies of *T. indotineae* and *A. verrucosus* were more floccose and showed buff to yellowish-brown coloration on the reverse side (Fig. 3A). The light microscopy findings (Fig. 3B) were also confirmed by scanning electron microscopy (SEM) (Fig. 3C), showing spherical conidia on occasional long stalks in *M. keratinophilum* as compared to tear-shaped conidia borne directly on the hyphae in *T. indotineae*. Furthermore, *A. verrucosus* showed clavate lateral conidia borne directly on the hyphae. *M. keratinophilum* possessed smaller conidia (~2 µm; Fig. 3C) as compared to *A. verrucosus* (~8 µm; Fig. 3C) and *T. indotineae* (~6.5 µm; Fig. 3C). All three species showed confluent growth at 27°C within 10 days. Notably, moderate growth was also observed at 37°C for all three species.

### Antifungal susceptibility testing

*In vitro* antifungal susceptibility data for all the isolates (*n* = 64), by the Clinical and Laboratory Standards Institute broth microdilution method (CLSI-BMD), using the M38, 3rd edition, have been summarized in Table 1. Notably, high minimum inhibitory concentration (MIC) values of ≥16 mg/L were reported for both griseofulvin (GRI) and itraconazole (ITC) in all the *M. keratinophilum* isolates and three of the *A. verrucosus* isolates. However, high MIC values for terbinafine (TRB) against *T. indotineae* (MIC ≥ 0.5 mg/L) were observed in seven isolates (5). As no MIC cut-offs are presently available for several keratinophilic fungi, a comparison of geometric mean MIC (GM MIC) was made against TRB, which revealed a higher GM MIC of 0.617 mg/L for *T. indotineae* as compared to *M. keratinophilum* (GM MIC 0.344 mg/L) and *A. verrucosus* (GM MIC 0.279 mg/L). Furthermore, GM MICs against ketoconazole (KTC) and voriconazole (VRC) for *M. keratinophilum* (2.658 and 0.309 mg/L, respectively) were high as compared to the GM MICs for *A. verrucosus* (0.115 and 0.100 mg/L, respectively) and *T. indotineae* (0.994 and 0.145 mg/L, respectively). Olorofim (OLO; MIC range ≤ 0.003–0.25 mg/L) and luliconazole (LUZ; MIC range ≤ 0.003–0.03 mg/L) showed excellent activity against all three species. Overall, *M. keratinophilum* showed reduced susceptibility toward all the azoles tested as compared to the other two species.

**TABLE 1:** *In vitro* antifungal susceptibility profile of 64 isolates against 10 antifungal drugs by using broth microdilution method (CLSI M38, 3rd edition)

Drug^*a*^	Species^*f*^	No. of isolates with MIC^*b*^ (mg/L) of:																	Range	GM-MIC^*c*^	MIC-50^*d*^	MIC-90^*e*^
		0.003	0.007	0.015	0.03	0.06	0.125	0.25	0.5	1	2	4	8	16	32	64	128	256				
TRB	M.k.					1	4	19	6	9									<0.06-1	0.344	0.25	1
	A.v.					2	2	2	4	2									<0.06-1	0.279	0.25	1
	T.i.					1	3	2	1	2		3	1						<0.06-8	0.617	0.5	4
GRI	M.k.													39					>16	16	16	16
	A.v.						4	3	2					3					0.125- > 16	0.629	0.25	16
	T.i.									6	3	1		3					1- > 16	2.475	2	16
ITC	M.k.													39					>16	16	16	16
	A.v.				5	1		3						3					<0.03- > 16	0.259	0.06	16
	T.i.				2	3	1	3	4										<0.03-0.5	0.152	0.25	0.5
VRC	M.k.						4	20	14	1									0.125-1	0.309	0.25	0.5
	A.v.				5	1	2	2	1	1									<0.03-1	0.100	0.06	0.5
	T.i.				2	1	4	4	2										<0.03-0.5	0.145	0.125	0.5
FLC	M.k.												1			8	22	8	8- > 256	119.216	128	256
	A.v.									5			3	1			2	1	1- > 256	7.551	8	128
	T.i.											1	3	1	3	4	1		4-128	25.853	32	64
ISA	M.k.						2	2	9	18	8								0.125-2	0.822	1	2
	A.v.			3		2			4	1	2								<0.015- 2	0.184	0.5	2
	T.i.				1		2	2	3	5									<0.03-1	0.382	0.5	1
POS	M.k.				1		3	11	21	3									0.03-1	0.362	0.5	0.5
	A.v.			4		1	4	3											<0.015- 0.25	0.069	0.125	0.25
	T.i.				4		1	4	3		1								<0.03-2	0.17	0.25	0.5
KTC	M.k.										23	16							2-4	2.658	2	4
	A.v.				4	3	2		1		2								<0.03-2	0.115	0.06	2
	T.i.				1	1	1	1	1		4	2	1	1					<0.03- > 16	0.994	2	8
LUZ	M.k.	12	15	7	5														<0.003-0.03	0.007	0.007	0.03
	A.v.	9	1	2															<0.003-0.015	0.004	0.003	0.015
	T.i.	8		5															<0.003-0.015	0.005	0.003	0.015
OLO	M.k.	16	8	12	3														<0.003-0.03	0.007	0.007	0.015
	A.v.	5		3	1	1	1	1											<0.003-0.25	0.013	0.015	0.125
	T.i.	5	3	4	1														<0.003-0.03	0.007	0.007	0.015

^*a*^ TRB, terbinafine; GRI, griseofulvin; ITC, itraconazole; VRC, voriconazole; FLU, fluconazole; ISA, isavuconazole; POS, posaconazole; KTC, ketoconazole; LUZ, luliconazole; OLO, olorofim.

^*b*^ MIC, minimum inhibitory concentration.

^*c*^ GM-MIC, geometric mean minimum inhibitory concentration.

^*d*^ MIC50, MIC at which growth of 50% tested isolates was inhibited.

^*e*^ MIC90, MIC at which growth of 90% tested isolates was inhibited.

^*f*^ M.k., *Myriodontium keratinophilum*; A.v., *Aphanoascus verrucosus*; T.i., *Trichophyton indotineae*.

### The draft (hybrid *de novo*) genome assembly of *M. keratinophilum*

We present the first hybrid *de novo* genome assembly of *M. keratinophilum* isolate D16, along with seven Illumina assemblies. The genome of *M. keratinophilum* is 24,768,318 bp, with a BUSCO completeness of 97.2%. The hybrid assembly, with 29 contigs, compared to the Illumina assemblies (104 to 118 contigs), suggests a comparatively complete genome. *M. keratinophilum* with 6,766,630 bp largest contig and 9 other contigs (L50+L90) of size >1.3 Mb (N50+N90) shows less fragmentation of the genome (Table 2). Overall, the GC content (47.07%–47.25%) and the BUSCO completeness (95.4%–97.2%) of all the *M. keratinophilum* assemblies were comparable (Table S2).

**TABLE 2:** Comparative genome assembly statistics and *ab initio* annotation features

	*Myriodontium* *Keratinophilum* MK16 (hybrid assembly^a^)	*Aphanoascus verrucosus* AV4^b^	*Trichophyton indotineae* TI51^b^
Number of contigs	29	360	317
Total length	24,768,318	23,046,821	21,990,989
Largest contig (bp)	6,766,630	1,505,050	461,592
GC contents (%)	47.08	49.58	48.69
N50 (bp)	3,464,938	432,428	127,025
N90 (bp)	1,390,692	157,370	40,028
L50	3	17	52
L90	6	49	168
# N’s per 100 kbp	0.00	0.00	0.00
BUSCO completeness (%)	97.2	98	98.7
*Ab initio* predicted protein coding regions	9,577	8,286	8,494
Number of tRNA coding regions	102	132	95
Number of rRNA coding regions	25	25	16

^a^ The hybrid assembly was made using both Nanopore and Illumina reads.

^b^ Illumina assembly.

### *Aphanoascus verrucosus* genome assembly

We generated seven *de novo* assembled genomes of *A. verrucosus* in the current study and compared them with the available assembly on NCBI (accession: GCA_014839905.1, strain: IHEM 4434). We generated a 23.04 Mb genome with contig-level assembly, similar to the available genome. Further assessment showed improved BUSCO completeness, with 98% single-copy genes (94.7% in the reference IHEM 4434), the largest contig >1.3 Mb across five isolates, N50 up to 444 kbp, and zero unassigned bases (N) per 100 kbp. GC content of all the assemblies (49.5% to 49.6%) was comparable (Table S2).

### Comparative genome assembly of *M. keratinophilum, A. verrucosus,* and *T. indotineae*

Overall, genome sequences of 18 isolates detailed in Table 3 were analyzed. The Illumina assembly statistics of AV4 and TI51 isolates of *A. verrucosus* and *T. indotineae,* with fewer contigs and/or the highest BUSCO completeness, were compared with *M. keratinophilum* MK16 and are detailed in Table 2. The total lengths of the three assemblies analyzed (*M. keratinophilum*, *A. verrucosus,* and *T. indotineae*) are 24.76 Mb (MK16), 23.04 Mb (AV4), and 21.99 Mb (TI51). The number of unassigned bases (N) per 100 kbp was zero in all these assemblies, which is a significant improvement on the currently available Illumina-assembled whole-genome data (*A. verrucosus*: IHEM 4434). Our analysis predicted 102, 132, and 95 tRNA coding genes (including a few pseudogenes) in *M. keratinophilum*, *A. verrucosus*, and *T. indotineae*, respectively. While the number of rRNAs was 25 in both *M. keratinophilum* and *A. verrucosus*, it was 16 in *T. indotineae*. GC content in the *A. verrucosus* genome was marginally higher (49.58%) compared to the other two genera. These three isolates (MK16, AV4, and TI51) were further used as the reference for comparative analysis of *M. keratinophilum*, *A. verrucosus*, and *T. indotineae*, respectively, in this study.

**TABLE 3:** Details of isolates sequenced in the current study

Species	Isolate ID	Sample source	MIC^*a*^									
			TRB	GRI	ITC	FLU	VRC	ISA	POSA	KETO	LUZ	OLO
*Myriodontium keratinophilum*	D2	Nail	0.5	>16	>16	128	0.25	1	0.5	4	0.015	0.015
	D4	Skin	0.25	>16	>16	128	0.25	1	1	2	0.007	0.015
	D5	Hair	0.125	>16	>16	64	0.25	0.5	0.25	2	<0.003	<0.003
	D8	Nail	0.25	>16	>16	128	0.125	0.125	0.5	2	<0.003	0.007
	D13	Skin	0.25	>16	>16	64	0.125	1	0.25	2	<0.003	0.015
	D16/MK16	Hair	1	>16	>16	>256	0.5	1	0.5	4	0.015	0.015
	D52	Skin	0.25	>16	>16	128	0.25	1	0.5	2	0.015	0.015
	D89	Skin	0.5	>16	>16	128	0.5	1	0.25	4	0.03	0.007
*Aphanoascus verrucosus*	D1/AV1	Nail	1	0.125	0.25	8	0.125	0.015	0.125	0.125	<0.003	<0.003
	D43/AV4	Skin	0.125	>16	>16	>256	1	1	0.25	<0.03	<0.003	0.06
	D47/AV5	Skin	<0.06	0.5	<0.03	1	<0.03	0.06	<0.015	<0.03	<0.003	0.015
	D55/AV6	Hair	<0.06	0.5	<0.03	1	<0.03	0.5	<0.015	<0.03	<0.003	0.125
	D63/AV7	Skin	0.25	0.125	<0.03	1	<0.03	0.5	<0.015	0.06	<0.003	0.015
	D64/AV8	Hair	0.5	0.25	<0.03	1	0.06	0.5	<0.015	0.06	<0.003	0.25
	D70/AV9	Nail	1	0.125	0.25	8	0.125	0.015	0.125	0.125	<0.003	<0.003
*Trichophyton indotineae*	D37/TI37	Skin	4	1	<0.03	8	<0.03	<0.03	0.25	<0.03	<0.003	<0.003
	D51/TI51	Skin	0.25	>16	0.5	64	0.5	1	0.25	4	0.015	0.015
	D57/TI57	Nail	<0.06	1	<0.03	16	0.25	0.125	0.125	0.125	<0.003	<0.003

^*a*^ MIC, minimum inhibitory concentration; TRB, terbinafine; GRI, griseofulvin; ITC, itraconazole; VRC, voriconazole; FLU, fluconazole; ISA, isavuconazole; POS, posaconazole; KTC, ketoconazole; LUZ, luliconazole; OLO, olorofim.

### Ortholog phylogenetic tree confirms a close genetic relationship of *M. keratinophilum* and *A. verrucosus* with *Arthrodermataceae* and *Onygenaceae*, respectively

**Fig 4:**
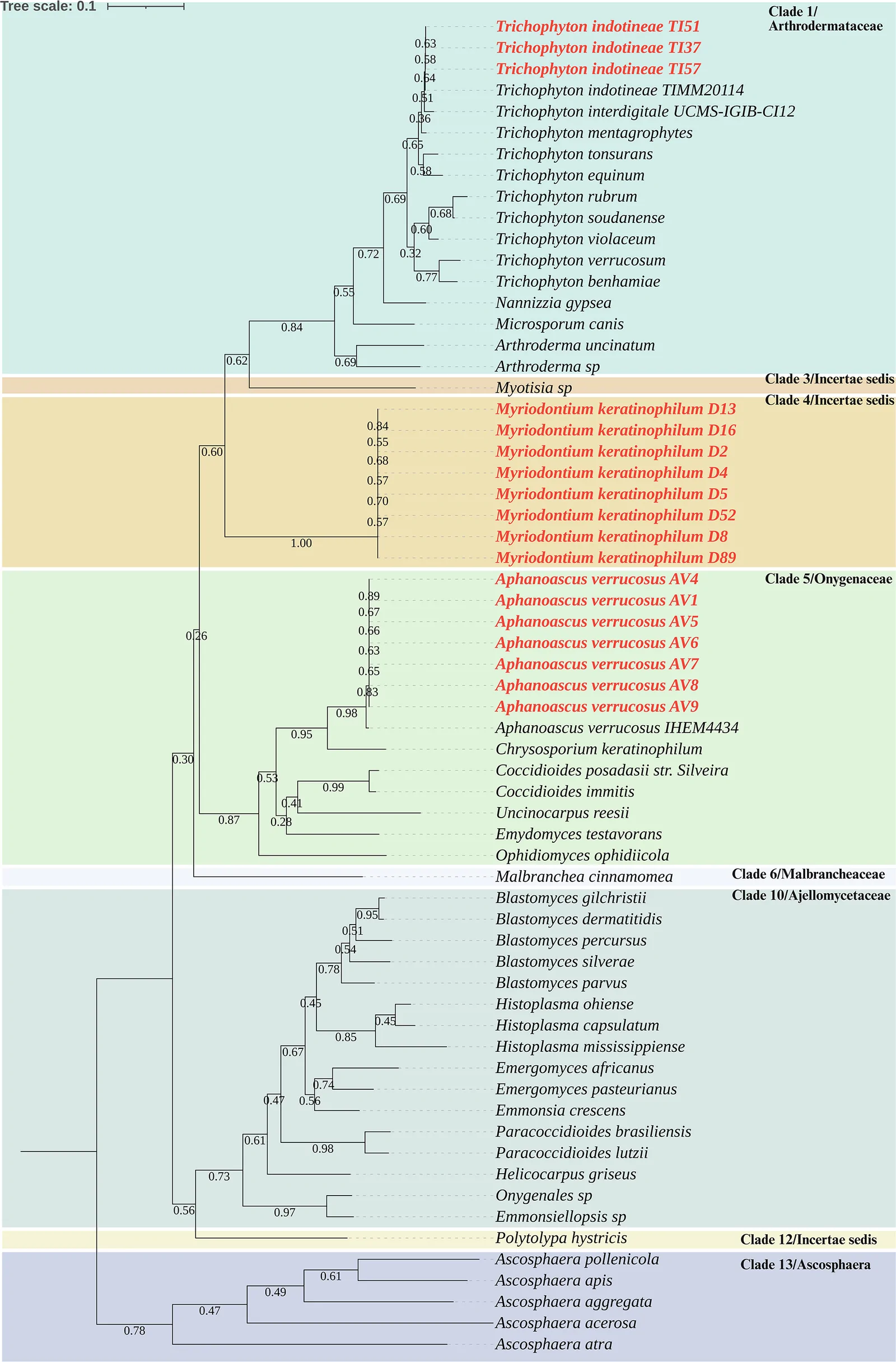
Ortholog phylogenetic tree built on 10,242 orthogroups, including 95.2% of the total genes from 46 members of the order Onygenales. *M. keratinophilum* (*n* = 8), A. *verrucosus* (*n* = 7), and *T. indotineae* (*n* = 3) from the present study are shown in red. Branch length (shown as tree scale) is the distance of a species from the point of divergence. Branch values (0.20–1) indicate the bootstrap (20–100) of the respective clade.

Ortholog-based phylogeny placed all the species (*n* = 46) of order Onygenales in eight clades (Fig. 4), as described by Kandemir et al. (24) using multigene phylogeny. Isolates of *M. keratinophilum* formed a separate clade (clade 4, *Incertae sedis*) with a bootstrap of 1.00 and are comparatively closer to *Arthrodermataceae* than *Onygenaceae*. Interestingly, *A. verrucosus* isolates from the present study, along with the IHEM strain, clustered together under Group 5-II as previously classified by Kandemir et al. Analysis showed *T. indotineae* is closely related to the TIMM20114 reference strain of *T. indotineae* in clade 1, followed by *Trichophyton interdigitale*, *Trichophyton mentagrophytes*, *Trichophyton tonsurans*, *Trichophyton equinum*, and other *Trichophyton* species.

### *M. keratinophilum* and *A. verrucosus* show close intra-species genetic relatedness

The variant calling by Snippy identified a median of 100 (range: 27 to 151) and 27 (range: 18 to 53) modified bases, including single nucleotide polymorphisms (SNPs), insertions, deletions, and complex modifications in *M. keratinophilum* and *A. verrucosus* isolates, respectively (Table S3). Notably, such a small difference within a 23 to 24 Mb genome shows a close genetic relationship among the isolates of a selected species. Of the three *T. indotineae* isolates sequenced, TI57 showed 84 modifications compared to isolate TI37 (352 modified bases) in its genome when mapped to the reference *T. indotineae* TI51 isolate.

### Functional annotation of *M. keratinophilum*, *A. verrucosus*, and *T. indotineae*: evidence of more coding regions in *M. keratinophilum*

Despite the availability of genome assemblies of *T. indotineae* and *A. verrucosus* on NCBI database, no annotation has been done to date. Annotation of all the three keratinophilic fungi was done in the present study. The structural comparisons among the three species have been done using the best assembled genome (comparatively complete) from each species, i.e., MK16, AV4, and TI51. *Ab initio* structural annotation of the draft assemblies predicted over 9,577 protein-coding regions in the *M. keratinophilum* genome (Table 2), whereas the other two fungi showed comparatively fewer protein-coding regions, i.e., 8,286 in *A. verrucosus* and 8,494 in *T. indotineae* (details of annotated genes for the three species can be found in Table S4). Mapping these newly identified coding sequences using the BlastKOALA web annotation service to the KEGG eukaryotic gene database resulted in functional annotation of 3,956 (41%), 3,694 (45%), and 3,793 (45%) genes in *M. keratinophilum*, *A. verrucosus*, and *T. indotineae*, respectively. The KEGG mapper categorized these functionally annotated genes into 6 pathways, which were further sub-categorized into 22 KEGG categories. Among the protein-coding genes within a genus, the majority were linked to two categories: metabolism (63%–64%) and genetic information processing (21%–22%). Though the proportion of genes assigned was not significantly different among the three species, the number of genes involved in carbohydrate metabolism was comparatively higher in *M. keratinophilum* (*n* = 311) than in the other two fungi (*n* = 257 in *A. verrucosus* and *n* = 258 in *T. indotineae*).

### Identification of carbohydrate-active enzymes with unique substrate affinities and the subtilisin 3 gene in *M. keratinophilum*

**Fig 5:**
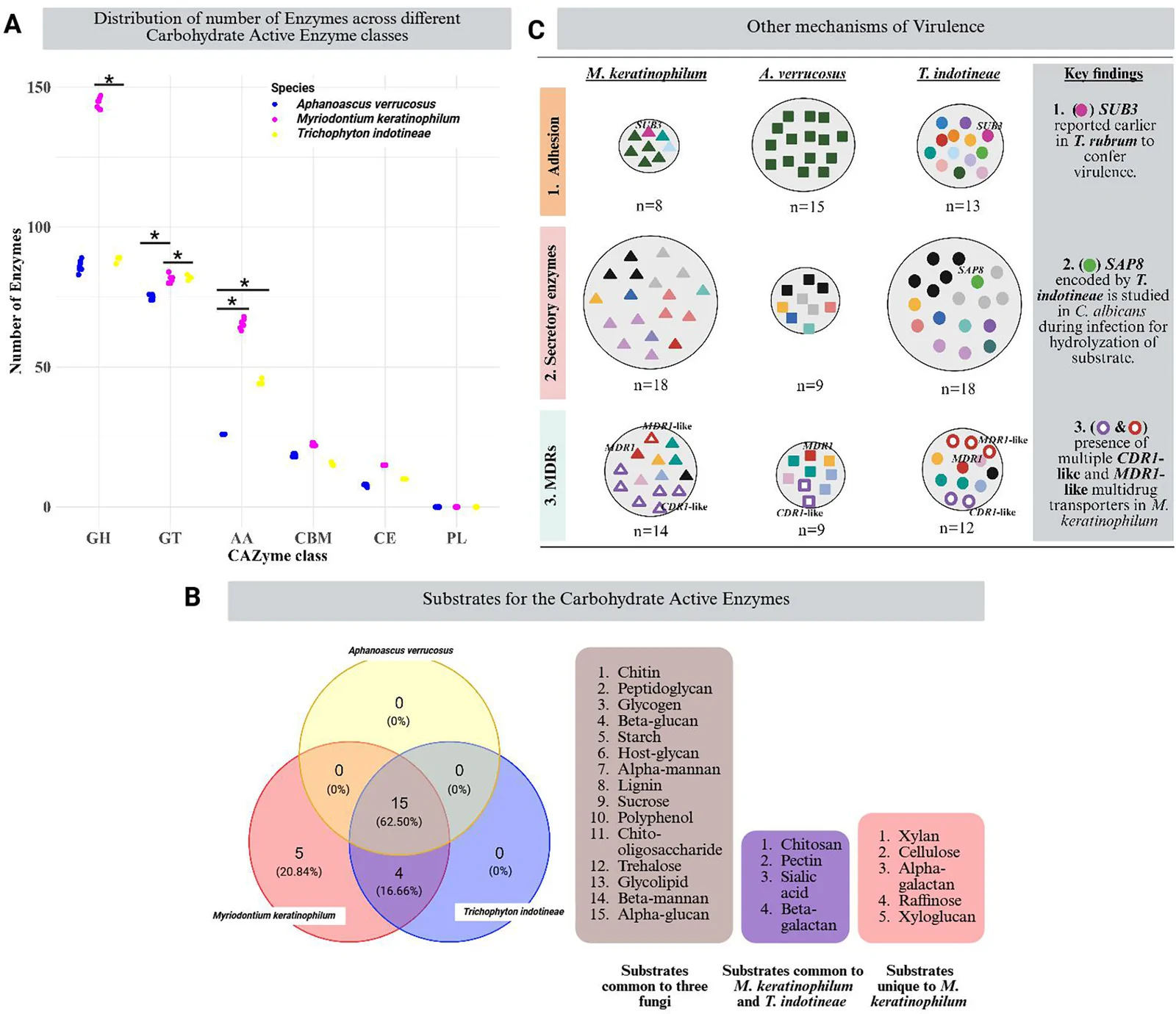
(**A**) CAZyme classes and numbers: GH, glycoside hydrolases; GT, glycosyltransferases; AA, auxiliary activities; CBM, carbohydrate-binding module; CE, carbohydrate esterase; PL, polysaccharide lyase. (**B**) List of substrates for carbohydrate-active enzymes in three species. (**C**) The number of genes in three categories (adhesion, secretory enzymes, and multidrug resistance transporters [MDRs]) encoded is indicated by the size of the circle. The virulence genes encoded by *M. keratinophilum*, *A. verrucosus*, and *T. indotineae* are shown as shaded triangles, squares, and circles, respectively. Different colors represent unique genes within a particular category (color code and the respective gene name are detailed in Table S5). *P value < 0.0005.

The genes involved in the carbohydrate metabolism pathway (carbohydrate-active enzymes [CAZymes]) have previously been reported to have a role in determining host specificity and virulence. In keeping with the results of KEGG functional annotation, a comparatively higher number of CAZyme families were identified in *M. keratinophilum* (646) than in *A. verrucosus* (491) and *T. indotineae* (541) using dbCAN3; the number of CAZymes is calculated from the reference isolates (MK16, AV4, and TI51) for the three species. All these CAZyme families were further categorized into five classes (Fig. 5A), among which the glycoside hydrolases (GH) are most prevalent in all three species, followed by glycosyltransferases (GT), auxiliary activities (AA), carbohydrate-binding modules (CBM), and carbohydrate esterases (CE). In contrast, none of the three species encodes enzymes assigned to the sixth CAZyme class, polysaccharide lyase (PL). Notably, our analysis indicates that among the CAZymes, the proportion of GH enzymes was significantly higher in *M. keratinophilum* isolates compared to the well-known dermatophyte *T. indotineae* isolates (GH: 44% vs. 36%; *P* < 0.0005; Fig. 5A). The proportion of GT enzymes was significantly higher in *T. indotineae* than in *M. keratinophilum* (GT: 34% vs. 24%; *P* < 0.0005; Fig. 5A). Notably, both *M. keratinophilum* and *T. indotineae* had a higher proportion of AA enzymes than *A. verrucosus* (AA: 19%, 18% vs. 12%; *P* < 0.0005; Fig. 5A).

Subsequent substrate prediction for these five CAZyme families by HMMER search using the dbCAN-sub HMM database resulted in the identification of substrates for 51% (330/646), 44% (214/491), and 44% (240/541) of enzymes in *M. keratinophilum*, *A. verrucosus*, and *T. indotineae*, respectively (Table S4). Unlike the other two species, CAZymes in *M. keratinophilum* are highly diverse, including enzyme families with five unique substrate affinities (xylan: CBM13 and CE4, cellulose: AA9, raffinose: GH27, alpha-galactan: GH27, and xyloglucan: GH95; Fig. 5B). Furthermore, both *M. keratinophilum* and *T. indotineae* share the following three CAZyme families: GH75, GH2, and GH43, with affinity to degrade chitosan and beta-galactan. Additionally, *M. keratinophilum* has unique CAZyme GH35 for the substrate pectin, in addition to GH33, which is also present in *T. indotineae* (Table S4). Unlike *M. keratinophilum* and *T. indotineae*, *A. verrucosus* does not code for any of these CAZymes (*n* = 9; Fig. 5B). The diverse substrate specificity of *M. keratinophilum* and *T. indotineae* supports their ability to thrive in diverse environments outside the human host and infect non-human hosts.

Based on previous reports, the classical virulence mechanisms among dermatophytes include (i) adhesion genes, (ii) secretory enzymes, and (iii) multidrug resistance transporters (MDRs). The adhesion genes primarily include subtilisin (*SUB*)-like proteases, which help the pathogen to bind to the epidermis. The total number of adhesion genes (including copies of the same gene) encoded by *M. keratinophilum* (*n* = 8) and *T. indotineae* (*n* = 13) is less than that of *A. verrucosus* (*n* = 15). However, it is important to note that the *SUB3* gene, which is known for fungal pathogenicity, is present in both *M. keratinophilum* and *T. indotineae* (Fig. 5C). Interestingly, when we analyze the number of unique adhesion genes, *T. indotineae* has the highest number, i.e., 13 unique genes out of a total of 13 genes, followed by *M. keratinophilum,* i.e., 4 unique genes out of 8 (Fig. 5C). *A. verrucosus*, on the other hand, lacked commonly known subtilisin genes (*SUB1–SUB12*), showing the least diversity in terms of unique adhesion genes and possessed 15 copies of a single *SUB* gene, CPC 735 (Fig. 5C).

We analyzed several secretory enzymes, including secretory aspartic proteases (*SAP*) (Table S5). Notably, *SAP8* (Fig. 5C), known for substrate hydrolysis during infection, is present in *T. indotineae*. Furthermore, *M. keratinophilum* and *T. indotineae* code for twice the number of secretory enzymes (*n* = 18) compared to *A. verrucosus* (*n* = 9). Membrane-embedded efflux pumps modulate susceptibility and virulence by active efflux of the antifungals and compounds of similar structure. These membrane-embedded efflux pumps are mostly ATP-binding cassette (ABC) transporters. In dermatophytes, these ABC transporter encoding genes are commonly named *MDR* genes and the *Candida* drug resistance 1 (*CDR1*) gene due to their sequence similarity with known genes in several *Candida* spp*. M. keratinophilum* codes for the maximum number of *MDRs* (*n* = 14, *n* = 9 for *A. verrucosus,* and *n* = 12 for *T. indotineae*; Fig. 5C). Specifically, *M. keratinophilum* has multiple *CDR1*-like genes (*n* = 6) compared to the other two species (*n* = 2 in each species; Fig. 5C). Notably, *M. keratinophilum* and *T. indotineae* carry all the classical *MDR* genes known to be present in dermatophytes, i.e., *MDR1*, *MDR2*, *MDR3*, *MDR4*, and *MDR5*, while *A. verrucosus* lacks *MDR4* (Table S5). Furthermore, both *T. indotineae* and *M. keratinophilum* have at least one *MDR1*-like gene (Fig. 5C).

The *CDR1*-like and *MDR1*-like genes are *de novo* predicted protein-coding regions that have 40%–70% sequence similarity with the *CDR1* and *MDR1* genes, respectively (Table S5). While *M. keratinophilum* has all the genes encoded by highly virulent *T. indotineae*, *A. verrucosus* lacks *MDR4* in its genome and has the lowest number of *MDR* genes.

### Functional annotation predicts two paralogs of *CYP51* (*A* and *B*) in *M. keratinophilum* and *A. verrucosus*

Sterol 14-alpha demethylase is the azole target and has two isoforms in dermatophytes (*CYP51A/B*). While *CYP51 B* is common to all members of Ascomycota, the other paralogs, *CYP51A* and/or *CYP51C*, often vary among individual species. Functional annotation of *ab initio* predicted protein-coding regions identified two paralogs (named P1 and P2) of *CYP51* in all the *M. keratinophilum* and *A. verrucosus* isolates. A sequence-based phylogenetic analysis of the two *CYP51* enzymes was performed. Our analysis showed an increased divergence of the two paralogs; P1 has sequence similarity to *CYP51B*, and P2 has sequence similarity to *CYP51A* (Fig. S3). Of note, none of the sequences from the three fungi sequenced in this study contained paralogs similar to *CYP51C*.

Among the three isolates of *T. indotineae*, TI37 harbored an amino acid substitution Y444H in the *CYP51* gene for *T. indotineae* isolate TI37 (ITR MIC < 0.03 mg/L; Table 1) and F397L in squalene epoxidase (*SQLE*) gene (TRB MIC 4 mg/L; Table 1). Results of the tandem repeat finder showed the absence of repeats in 1 kbp upstream of the *CYP51* and *SQLE* genes in all three species.

## DISCUSSION

This study documents the association of two rare keratinophilic fungi, *M. keratinophilum* and *A. verrucosus*, with dermatophytosis in a cohort of patients with superficial fungal infections from the Kashmir region of North India. In addition, comparative genome characterization and annotation of these two fungi, together with *T. indotineae*—a recently identified pathogen responsible for widespread and recalcitrant dermatophytosis globally—revealed differences in key molecular determinants implicated in adhesion, antifungal resistance, and putative virulence among these fungi. Interestingly, both *M. keratinophilum* and *A. verrucosus* have been reported from diverse environmental sources; however, neither of the keratinophilic fungi was recovered from garden soil samples in the present study. Nevertheless, isolation of the related keratinophilic species, *Aphanoascus terreus*, from a shared public space within the garden suggests the possibility of common environmental exposure to keratinophilic fungi. The current study is primarily laboratory-based and did not collect data on socioeconomic determinants; consequently, the role of environmental factors in the acquisition of *M. keratinophilum* and *A. verrucosus* among patients with dermatophytosis remains undetermined.

Recent genome-wide sequencing of *T. indotineae* isolates collected from 14 countries revealed a median of 106 SNPs (range: 0–392) among the samples. The minimal genetic variation observed among *T. indotineae* isolates across these countries supports the clonal dissemination and swift global transmission of *T. indotineae* (27). In the present study, SNP analysis of *M. keratinophilum* and *A. verrucosus* isolates demonstrated low intra-species genomic diversity, with 100 (range: 27–151) and 27 (range: 18–53) base modifications, respectively, suggesting high relatedness of strains among patients. Furthermore, future transmission in other parts of India, as seen with *T. indotineae*, is possible, requiring molecular identification of dermatophytes, crucial for accurate species identification. This study presents the first complete genome assembly and annotation for *M. keratinophilum*. Additionally, we provide an improved and comparatively comprehensive genome for *A. verrucosus* relative to the previously published genome (*A. verrucosus* IHEM 4434, Biosample-SAMN15800566). Structural and functional annotations of both species, compared with annotated *T. indotineae*, indicate the presence of the *SUB3* gene in *M. keratinophilum* and *T. indotineae*. *SUB* not only contributes to keratin degradation but also facilitates adhesion to keratinocytes. The increased expression of *SUB* in infected reconstructed human epidermis models during early infection suggests an important role in adhesion (28). Most dermatophytes contain 12 *SUB* genes (*SUB1–SUB12*), of which *SUB3* and *SUB6* are two important virulence factors (29–31). In an *ex vivo* study, when arthroconidia from the *SUB3* RNA-silenced *Microsporum canis* strain were introduced to the feline adherence model, they showed reduced adherence to feline epidermis compared to the control strain (32). Furthermore, a quantitative study on virulence factors in *Trichophyton benhamiae*, infecting a guinea pig skin explant-based *ex vivo* model, showed increased transcript levels of the *SUB3* gene compared to *SUB6* and other genes (31). The key characteristic feature of the dermatophyte genome is the presence of carbohydrate-binding lysin motif (LysM) domain-containing proteins, which are present in all three fungi identified in this study. These LysM domain-containing proteins facilitate adhesion between fungal cell wall chitin and human skin glycoproteins during infection, further masking chitin and fungal glycoproteins, impeding innate immune recognition, and thus downregulating the host immune response (33). It is important to emphasize that functional validation and mechanistic studies of several key genes, identified in these fungi, are warranted to establish the potential role of these determinants in pathogenesis and adaptation in future studies.

Comparative genomic analysis reveals common enzymes in *T. indotineae* and *M. keratinophilum*, including enzymes that act on substrates such as chitosan, beta-galactan, sialic acid, and pectin. Sialidases have a role in maintaining cell wall integrity. The deletion of a sialidase-encoding gene in *Aspergillus fumigatus* resulted in chitin deposition and unusual hyphal morphology, highlighting the role of this enzyme in maintaining cell wall integrity (34). Notably, enzymes that degrade chitosan might be helpful for the fungi, as chitosan is reported to be a potent antifungal against many fungal species, including *Trichophyton rubrum* (35). The CAZymes are known to play a crucial role in fungal virulence (36, 37). In the present study, we have identified CAZymes: AA9, CBM13, CE4, GH95, GH75, and GH2 in *M. keratinophilum* that can degrade cellulose, xylan, xyloglucan, and chitosan, which are important components of the plant cell wall. The presence of these genetic determinants in *M. keratinophilum,* which survives in soil and is a known entomopathogenic fungus, is important for inhabiting specific ecological niches or host ranges and for survival in the environment. Interestingly, previous comparative genomic analysis of *T. rubrum* and related dermatophytes reported that several CAZyme classes are depleted in dermatophytes, including GH families involved in the breakdown of hemicellulose or pectin, major components of the plant cell wall (30). The dermatophyte genomes do not contain any identifiable cellulose-binding domains, which are typically a hallmark of fungi that utilize plant material for nutrients (30).

Similar to *T. indotineae*, *M. keratinophilum* harbored unique GH family genes, demonstrated growth at 37°C, and was isolated in culture from dermatophytic lesions, suggesting its possible association with dermatophytosis cases presenting as tinea corporis and tinea cruris. It is pertinent to emphasize that in the Indian subcontinent, with a high burden of *T. indotineae* infections, and also in high-resource settings, most cases of dermatophytosis in outpatient clinics are diagnosed clinically. The treatment outcome and follow-up data were not recorded; however, the majority of tinea corporis and cruris patients received oral TRB for 6 to 8 weeks, while in recurrent dermatophytosis cases (5%), oral ITC was given for 4 to 6 weeks. Also, in 5% of cases of tinea corporis and tinea cruris with small skin lesions, topical application of antifungals, clotrimazole, or KTC, was applied. Importantly, in the present study, *T. indotineae* and *A. verrucosus* were cultured from 20% (*n* = 13) and 19% (*n* = 12) of dermatophytosis cases. Both species require ITS region sequencing for definitive identification, the facilities for which are lacking in routine microbiology laboratories. It is important to emphasize that mycology laboratories should remain vigilant regarding the accurate identification of dermatophytes and prepare to implement molecular identification of dermatophytes in the current dynamic scenario.

Notably, *M. keratinophilum* showed reduced *in vitro* susceptibility toward ITC, which is worrisome as ITC is the preferred antifungal against TRB-resistant *T. indotineae* infections, which are currently posing a therapeutic challenge. The emergence of *T. indotineae* in the last decade in the Indian subcontinent has been attributed to the misuse of over-the-counter combination creams, often containing potent corticosteroids, antibiotics, and antifungals that have contributed to the resistance. Additionally, it should be noted that region-specific climatic variables, such as increasing temperatures and xenobiotic pollutants, may partially explain the evolution of *T. indotineae* (38). Heat exposure may induce physiological changes in the fungal cell membrane, the target site for conventional antifungals, including TRB and ITC. Furthermore, selection pressure for efflux pumps against xenobiotics may also confer antifungal resistance. The primary efflux pumps are ABC transporters spanning the cell membrane. *M. keratinophilum* genome showed multiple *CDR1*-like genes, and additionally, *T. indotineae* and *M. keratinophilum* showed *MDR1*-like genes along with the classical MDR genes. In *T. rubrum*, ITC treatment induced transcription of *TruMDR2*, while the mutant strain (△*TruMDR2*) showed no difference in sensitivity compared to the control strain (39). Notably, many expression studies in *T. rubrum* in response to the antifungals and cytotoxic drugs have highlighted expressed sequence tags in some putative multidrug transporters or *MDR*-like or *CDR1*-like genes (40–42). This suggests their expression in response to these drugs and a possible role in modifying the pathogen’s phenotype. These additional forms of ABC transporters might have a compensatory role in the absence of the classical *MDRs*. While antifungal substrate alteration, such as point mutation in the *CYP51* gene and other ergosterol pathway genes, is specific, the expression of the ABC transporters is also reported to be altered due to non-antifungal compounds like ethidium bromide and 4-nitroquinoline N-oxide (4NQO) (43). Thus, it is important that for both the keratinophilic fungi associated with dermatophytosis in the present study, the function and transcriptional expression of *MDR*-like and *CDR*-like genes, and their role in infection models, are warranted to establish the association with antifungal drug resistance. The present study raises awareness regarding the rare opportunistic keratinophilic fungi and highlights their reduced susceptibility toward frontline antifungal drugs like ITC. Finally, clinicians and microbiologists should be aware of the various dermatophyte organisms that commonly cause tinea infections. Furthermore, adequate diagnosis and treatment of tinea infections will be a critical factor in reducing the number of antifungal-resistant dermatophyte strains.

## MATERIALS AND METHODS

### Clinical specimens and fungal isolates

A total of 109 patient samples were obtained over a period of 1 year (January to December 2021) from patients attending the dermatology outpatient department in a tertiary care hospital in Kashmir, North India. Scrapings of the lesions, nail clippings and hair were collected and processed for direct microscopic examination with a 10% KOH mount (40% for nail samples). The specimens were inoculated on two plates each of Sabouraud’s dextrose agar (SDA) containing gentamicin and chloramphenicol and the other containing cycloheximide (0.05%) and were incubated at 27°C for two weeks. Only the samples positive for both KOH mount and culture were further taken up for the study.

### Environmental isolate sampling

Soil samples and surface swabs of benches and swings from public parks were collected using sterile moist swabs. A total of 7 swabs were taken from benches and swings located in a single garden, and 15 soil samples were collected from two gardens. For soil samples, 1 g of soil was suspended in 9 mL of sterile 0.85% NaCl to get a 1:10 dilution. The suspension was vortexed thoroughly, and a 1:100 dilution was prepared after settling down for 20 min. A volume of 200 µL of this suspension was plated in triplicate on DTM (44) and PDA plates. The swabs were directly plated on fungal culture media including DTM, SDA, CHROM agar *Candida* plates (Becton, Dickinson, Baltimore, MD, USA), and PDA. The DTM and PDA plates were incubated for 2 weeks at 27°C, while SDA and CHROM agar plates were incubated for a week at 37°C. The plates were monitored every 24 h, and any fungal colony was sub-cultured for purification.

### Morphological examination and phenotypic identification

The preliminary morphological identification was done on PDA at 27°C for 14 days. For microscopic morphological details, slide cultures were prepared on PDA and mounted in lactophenol cotton blue. Additionally, the slide cultures were also prepared for SEM (Jeol, Japan; Zeiss, Germany). Briefly, the slide cultures were fixed in 6% glutaraldehyde for 6 h at room temperature. After washing, alcohol dehydration of the slide cultures was carried out by incubating them for 15 min in each of the increasing alcohol concentrations (20%–100%). The slides were dried, and imaging was undertaken.

### Molecular identification and phylogenetic analysis

Molecular identification of isolates was performed by sequencing the ITS region of the small subunit rDNA. Mycelium was harvested from 7- to 14-day-old pure cultures on PDA at 27°C and then subjected to bead beating in DNA extraction buffer (0.2 M Tris-HCl, 25 mM EDTA, 0.25 M NaCl, 0.5% SDS), followed by phenol, chloroform, and isoamyl alcohol (25:24:1) extraction and ethanol precipitation. The extracted DNA was amplified using the ITS-1 (5′-TCCGTAGGTGAACCTTGCGG-3′) and ITS-4 (5′-TCCTCCGCTTATTGATATGC-3′) primers (45). DNA sequencing was performed using the respective primers for PCR at a 0.5 μM concentration. All sequencing reactions were carried out in a 10-μL reaction volume using the BigDye Terminator kit version 3.1 (Applied Biosystems, Foster City, CA, USA), according to the manufacturer’s recommendations, and analyzed on an ABI 3130XL Genetic Analyzer (Applied Biosystems). ITS sequences were subjected to BLAST searches at GenBank (http://www.ncbi.nlm.nih.gov/BLAST/Blast.cgi). Sequence-based species identification was defined by ≥99% sequence similarity with ≥99% query coverage. The neotype and type strains of *M. keratinophilum* (CBS strains 947.73 and 256.81), *A. verrucosus* (WM07.130 and CBS strain 171.62), *Apinisia racovitzae* (CBS strain 156.77), along with strains of *Apinisia pyriformis*, were retrieved from GenBank and included for phylogenetic analysis. Sequences were aligned using Clustal X 2.1 software, and a phylogenetic tree of all ITS sequences was constructed using MEGA version 11 software with 1,000 bootstrap values (46).

### Antifungal susceptibility testing

Antifungal susceptibility testing (AFST) was carried out by CLSI-BMD, using the M38, 3rd edition (47). The isolates were grown for 7 days on PDA at 27°C, and conidial inoculum was prepared in saline containing Tween 80 by gently scraping the surface of mature colonies with a sterile cotton swab moistened with sterile physiological saline and allowed to settle. The final inoculum concentration was adjusted to 1 × 10^3^ to 3 × 10^3^ CFU/mL, counted by hemocytometer, which is double the concentration required for testing. Antifungals tested included allylamine, viz. TRB (Synergene India, Hyderabad, India), orotomide, viz. OLO (F2G Ltd.), triazoles, viz. ITC (Lee Pharma, Hyderabad, India), VRC (Pfizer Central Research, Sandwich, Kent, United Kingdom), fluconazole (FLC; Sigma-Aldrich, Germany), posaconazole (POS; Sigma-Aldrich, Germany), isavuconazole (ISA; Sigma-Aldrich, Germany); imidazoles, viz. LUZ (Emcure, Pune, India), KTC (Sigma-Aldrich), and GRI (Sigma-Aldrich). Drug-free and mold-free controls were included, and microtiter plates were incubated at 35°C. CLSI-recommended control strains of *Candida krusei* ATCC 6258 and *Candida parapsilosis* ATCC 22019 were included for every batch of isolates tested each day. MIC endpoints for all the drugs were defined as the lowest concentration that produced 80% inhibition of growth as read visually at 96 h.

### Temperature-based growth study

Growth patterns of species were studied at 27°C, 37°C, and 40°C. Inoculum was prepared as described earlier for AFST. Fifty microliters of the inoculum was plated in the center of the 90 mm PDA culture plate. The plates were incubated at the respective temperatures and observed every day for two weeks.

### Whole-genome sequencing

#### Short-read sequencing using Illumina platform

The details of isolates sequenced in the current study are presented in Table 3. For whole-genome sequencing (WGS), one isolate per patient was included. Overall, nine isolates from skin scrapings and nine isolates from nail and hair samples were selected. All isolates (*M. keratinophilum*, *n* = 8; *A. verrucosus*, *n* = 7; *T. indotineae*, *n* = 3) were sequenced on an Illumina HiSeq. DNA was extracted using a column-based method with a QIAamp DNA minikit (Qiagen, Hilden, Germany) and was quantified by QUBIT 3 Fluorometer using dsDNA HS Dye. WGS libraries were prepared using the NEBNext Ultra II DNA FS kit (New England Biolabs, Ipswich, MA, USA). In brief, 200 ng of intact DNA was enzymatically fragmented by targeting 200–300 bp fragment sizes, followed by purification using AMPure beads (Beckman Coulter Life Sciences, Indianapolis, IN, USA). For sequencing, the libraries were normalized to a 10 nMol/L concentration and pooled together at equal volumes. Furthermore, the library pools were denatured using freshly prepared 0.2 N NaOH for cluster generation on cBOT and sequenced on Illumina HiSeq 4000.

#### Oxford Nanopore sequencing

Long-read sequencing was done for *M. keratinophilum* (D16). Before DNA sequencing, the quality of the purified DNA was assessed using Implen NanoPhotometer N60, and integrity was checked on 0.8% agarose. For Oxford Nanopore sequencing, 1 µg of genomic DNA was processed using the Native Barcoding Kit SQK-NBD 112-24 and ligation sequencing kit SQK-LSK 112 according to the manufacturer’s protocol. A 100 ng (10–20 fmol) of the final DNA sample (average fragment size ~15 kbp) was loaded onto a FLO-MIN 112 version R10 flow cell and sequenced on a Gridion.

### Genome assembly

Fast5 files from Oxford Nanopore sequencing were basecalled using Guppy basecaller v6.5.7 using a high accuracy model (dna-r10.4_e8_hac.cfg) and an enabled adapter trimming option. For Illumina raw reads, adapter trimming was done using Trimmomatic v0.36 (48). The quality of both Oxford Nanopore basecalled reads and Illumina raw reads was checked using FastQC v0.11.9 and QualiMap v2.2.1. QC thresholds for Illumina raw reads were set to >30 (i.e., Phred score >30), and for Oxford Nanopore long reads, were set to >9. Unicycler v0.4.8 (49) with default assembler SPAdes v3.13.1, polisher Racon v1.4.20, and Pilon v1.24 were used to get the best *de novo* hybrid assembly using both Illumina short reads and Nanopore long read options. Furthermore, the genome polishing tool Pilon v1.24 (50) was used for Illumina assemblies to improve single-base resolution. These final polished assemblies generated by the polishing tool Pilon were subjected to removal of the contigs of length <200 bp. These final polished assemblies with contig length ≥200 were further assessed using Quast v5.0.2 (51) and BUSCO v5.6.1 (52) with Augustus as the default gene prediction tool and Onygenales_odb10 as the reference lineage data set in BUSCO. The mean depth for the assemblies was assessed using Samtools, and assemblies with >30× mean depth were included in the study.

### Variant calling and phylogeny

Variants, including insertions, deletions, SNPs, and complex variants, were detected using Snippy v4.6.0. *M. keratinophilum* (*n* = 8), *A. verrucosus* (*n* = 7), and *T. indotineae* (*n* = 3), sequenced in the present study, were analyzed along with 43 different species from the order Onygenales in the current phylogenetic analysis. The details of all 46 species of Onygenales whose whole-genome assembly and annotation data were available were downloaded and are given in Table S1. A phylogenetic tree was generated with a total of 10,242 orthogroups identified in all the assemblies of 46 species using Orthofinder v2.5.5 (53). The azole target *CYP51* gene paralog phylogenetic tree, including all strains of *M. keratinophilum* and *A. verrucosus*, was constructed using the maximum likelihood method and the default parameters of MAFFT v7.511 (54). Reference sequences of the *CYP51* gene homolog used in this phylogenetic analysis were obtained from the NCBI protein database (details of strains used are described in Table S1). The phylogenetic trees obtained were visualized using Interactive Tree of Life (iTOL) v6.9.1 (55) and annotated in BioRender.

### *Ab initio* structural, functional annotation, and analysis of antifungal-resistant genes in *M. keratinophilum* and *A. verrucosus*

We generated the first genome assembly of *M. keratinophilum* using both Nanopore long reads and Illumina short reads. Structural and functional annotation of all the dermatophytic species, i.e., *M. keratinophilum*, *A. verrucosus*, and *T. indotineae*, was done using BRAKER v3.0.8 (56) with inbuilt Augustus and GeneMark-ETP as *ab initio* gene predictors. The annotation of all the three species was done using all reference fungal amino acid sequences (8411689) available in the database OrthoDB v11 (57). The identified protein-coding regions were further functionally annotated using the BlastKOALA web annotation tool v3.0 (58). Both the carbohydrate enzymes and their substrates were identified by running the dbCAN3 annotation tool (59) using default parameters for HMMER: dbCAN, DIAMOND: CAZy, HMMER: dbCAN-sub, and dbCAN-PUL. As BlastKOALA only gives the pathway information, the genomes were further annotated using the UniProt database. The command line, database used, and the blast parameters used for functional annotation of the genomes are documented in Table S4. The genes known to confer virulence in the well-established dermatophytes were searched for their presence in the three fungi by keyword search, using the UniProt annotated data and BlastKOALA annotated data. To get the details of tRNA and rRNA, tRNAscan-SE v2.0.12 (60) and BrrnAP v0.9 were used, respectively. Previously known gene targets of antifungal drugs, TRB and ITC, were analyzed by multiple sequence alignment with MAFFT v7.511 (54) for the presence of single-nucleotide variants. Furthermore, tandem repeats in the upstream (up to 1 kbp) of the target gene were analyzed using Tandem Repeats Finder (TRF) v4.09.1.

## Data Availability

The genome sequences and the raw reads of all 18 isolates analyzed in the present study are submitted to the GenBank and the SRA database, respectively, under BioProject number PRJNA1451125. The WGS accession numbers for all the isolates sequenced are listed in Table S2.

## SUPPLEMENTAL MATERIAL

Supplemental figures spectrum.01087-26-s0001.docx Figures S1 to S3.

Table S1 spectrum.01087-26-s0002.xlsx Details of references and other isolates used in phylogenetic trees.

Table S2 spectrum.01087-26-s0003.xlsx Genome assembly statistics and data availability details of the isolates sequenced in the present study.

Table S3 spectrum.01087-26-s0004.xlsx Details of Single Nucleotide Polymorphisms (SNPs), including insertions, deletions, and complex modifications.

Table S4 spectrum.01087-26-s0005.xlsx Details of functional annotation of genomes: parameters used and annotated genes.

Table S5 spectrum.01087-26-s0006.xlsx Details of putative virulence genes, including multidrug resistance (MDR) transporter, adhesion, and secretory genes.

ASM does not own the copyrights to Supplemental Material that may be linked to, or accessed through, an article. The authors have granted ASM a non-exclusive, world-wide license to publish the Supplemental Material files. Please contact the corresponding author directly for reuse.
