# Supplementary material for: Dermatophytosis beyond Trichophyton indotineae: genomic characterization of rare keratinophilic fungi Myriodontium keratinophilum and Aphanoascus verrucosus in North India
Source: Microbiol Spectr. 2026 Sep 25;14(11):e01087-26. doi: 10.1128/spectrum.01087-26 (PMC13729104; doi:10.1128/spectrum.01087-26)
Supplement: Supplementary file 1 [file spectrum.01087-26-s0001.docx]

**Figure S1**. 10% KOH mounts showing fungal hyphae in samples of *A) Myriodontium keratinophilum*, B) *Aphanoascus verrucosus*, C) *Trichophyton indotineae*.

**Figure S2**: Phylogenetic tree based on the ITS region sequence showing clinical *Aphanoascus verrucosus* isolates and environmental *Aphanoascus terreus* isolate in one clade with *Myriodontium keratinophilum* isolates falling in a separate clade with 1000 bootstrap iterations (shown as 0.58-1 in tree).

**Figure S3**. CYP51 paralog phylogenetic tree for the three species. The isolates highlighted in red are from the present study, and in black are the reference strains. The tree scale is mentioned in the figure. Bootstrap 2000.
